# Optimizing Delivery of Mass Drug Administration for Schistosomiasis

**DOI:** 10.4269/ajtmh.19-0762

**Published:** 2019-10-28

**Authors:** Jennifer F. Friedman

**Affiliations:** 1 Center for International Health Research, Rhode Island Hospital, Providence, Rhode Island;; 2 Department of Pediatrics, The Warren Alpert Medical School of Brown University, Providence, Rhode Island

More than 200 million people are at risk for schistosomiasis globally. Despite the availability of safe and effective therapeutics, the optimal approach for treating those at risk remains unclear. Presently, the WHO guidelines recommend mass drug administration (MDA) with praziquantel in endemic communities, with a focus on reducing the prevalence of high-intensity infections. With this approach, individuals aged 4 years and older are offered treatment either in their communities or at schools, with the frequency of MDA determined by community prevalence of infection. Many gaps remain, however, with respect to the optimal frequency of MDA and the target populations within endemic communities.

In a report in this issue of the journal,^1^ collaborators from Kenya and Tanzania provide combined results from key studies funded by the Schistosomiasis Consortium for Operational Research and Evaluation (SCORE). These studies compared two different MDA approaches, examining the effects on infection intensity and prevalence as well as key markers of morbidity captured for children aged 7–8 years, who were followed up more than 5 years. Specifically, this substudy compared results for the most “extreme” schedules for treatment: community wide treatment (CWT) provided annually versus school-based treatment (SBT) provided every other year. Although results for each setting have been published previously, the comparison allowed greater power to detect differences and may also improve generalizability of findings.

After adjusting for baseline imbalances in infection prevalence and intensity, both the CWT and CBT groups demonstrated significant reductions in prevalence and intensity of infection, including heavy infections, by year five. The CWT group, which started with higher prevalence of infection, experienced a more significant decrease in prevalence than the SBT group. The decrease specifically in heavy infections is important as increased morbidity risk has been related to increased intensity of infection, and individuals with high egg outputs likely make a relatively greater contribution to the infection cycle.

Importantly, both groups had a residual prevalence of infection of approximately 45% at year five, reflecting a large remaining infection burden. This is likely to be even greater in the non-research context for many reasons. First, in the CWT arm, research staff actively sought out children who were attending school, using the school as a supplemental venue to capture them. Similarly, in the SBT arm, community mobilization teams encouraged children not attending school to report to the school for treatment. In addition, in the research setting, highly trained research staff may be better equipped to assuage fears and decrease treatment refusal in the CWT setting, where many barriers to uptake remain.^2^ These barriers include 1) concerns about treating women of reproductive age, 2) exclusion of children younger than 4 years who clearly experience infection and may be at greatest risk for morbidity, 3) individual-level refusal based on lack of knowledge regarding infection status, and 4) the misconception that schistosomiasis is not an important cause of morbidity and mortality.

With respect to morbidity outcomes, little difference between SBT and CWT was observed in this cohort of children. It should again be noted, however, that both arms actively attempted to treat all study subjects, minimizing the risk of missing treatments in this research context. However, there was variability in the change observed over time across morbidity markers in both groups. This was observed with stunting, with a significant increase in the prevalence of stunting between years one and five. This is to be expected and has been observed in observational studies,^3–5^ where each year a child living in an impoverished environment grows less than a child living in a better environment. It is possible that praziquantel positively impacted linear growth such that the progression of stunting may have been even more profound without the interventions, although this cannot be conclusively stated without the understandable lack of a control group. Similarly, although no significant changes were seen in either group with respect to anemia from baseline to 5 years, it remains possible that schistosomiasis treatment blunted an expected increase in anemia over time because of the cumulative effects of dietary iron insufficiency, blood loss due to hookworm infection, and menarche in some of the girls. These studies provide evidence that neither strategy was better with respect to anemia but, without a control group, does not allow inference with respect to whether these treatment strategies mitigated the risk of anemia over time.

A key finding from this study was the significant decrease in the prevalence of elevated portal vein diameter across both cohorts. This result supports the notion that provision of treatment at regular intervals can reverse or mitigate morbidity attributable to schistosomiasis. Other studies also demonstrated decreased portal vein diameter following treatment with praziquantel.^6,7^ This finding is important as portal vein diameter is much more specific for schistosomiasis than nutritional outcomes captured. Furthermore, portal vein diameter represents a marker for Symmers fibrosis, with consequent portal hypertension and risk of esophageal varices and gastrointestinal bleeding. Although it is reassuring that both treatment approaches decreased the prevalence of elevated portal vein diameter, it is also concerning that evidence of hepatic fibrosis was present among 9–11% of preadolescent children at baseline. Furthermore, given that the Niamey protocol, which compares values for portal vein diameter with a healthy reference population of similar height, was followed, it is unlikely that these changes in prevalence, which were highly significant, were confounded by age-related changes.

Findings from the SCORE studies emphasize the need for routine treatment for schistosomiasis, with positive effects using both strategies demonstrated particularly with respect to schistosomiasis-specific outcomes. These studies also highlight the need to do more given that even in this optimized research setting, the prevalence of infection remained unacceptably high in both groups after 5 years. It is important to note that the prevalence remained this high among the school-aged children who were actively targeted for treatment. It is expected that individuals living in communities assigned to SBT who do not attend school will remain infected and experience worsening morbidity over time. This includes preschool-aged children and school-aged children who cannot afford to attend school, pregnant women, and other adults. Rather than to choose either SBT or CWT, integrated approaches that maximize the number of treatments delivered should be used. Although we must consider costs of programs rolled out in resource poor settings, it is our role as physicians, scientists, and policy makers to first identify ideal approaches to minimize morbidity and mortality and advocate for the resources to meet these goals. This likely requires treatment more frequently than annually and across school, community, and clinical settings. Finally, as the world sees successes in decreasing the prevalence and intensity of infection, we will need to move from the current WHO focus on reduction in heavy-intensity infections toward the elimination of schistosomiasis.
